# A multistep genomic screen identifies new genes required for repair of DNA double-strand breaks in *Saccharomyces cerevisiae*

**DOI:** 10.1186/1471-2164-14-251

**Published:** 2013-04-15

**Authors:** Jennifer Summers McKinney, Sunaina Sethi, Jennifer DeMars Tripp, Thuy N Nguyen, Brian A Sanderson, James W Westmoreland, Michael A Resnick, L Kevin Lewis

**Affiliations:** 1 Department of Chemistry and Biochemistry, Texas State University, San Marcos, TX 78666, USA; 2 Laboratory of Molecular Genetics, National Institute of Environmental Health Sciences, NIH, Research Triangle Park, NC 27709, USA

**Keywords:** EcoRI, Homologous recombination, End-joining, Double-strand break, Bleomycin, MMS, Radiation, RAD52, Gene ontology (GO), Overlapping genes

## Abstract

**Background:**

Efficient mechanisms for rejoining of DNA double-strand breaks (DSBs) are vital because misrepair of such lesions leads to mutation, aneuploidy and loss of cell viability. DSB repair is mediated by proteins acting in two major pathways, called homologous recombination and nonhomologous end-joining. Repair efficiency is also modulated by other processes such as sister chromatid cohesion, nucleosome remodeling and DNA damage checkpoints. The total number of genes influencing DSB repair efficiency is unknown.

**Results:**

To identify new yeast genes affecting DSB repair, genes linked to gamma radiation resistance in previous genome-wide surveys were tested for their impact on repair of site-specific DSBs generated by *in vivo* expression of EcoRI endonuclease. Eight members of the RAD52 group of DNA repair genes (*RAD50*, *RAD51*, *RAD52*, *RAD54*, *RAD55*, *RAD57*, *MRE11* and *XRS2*) and 73 additional genes were found to be required for efficient repair of EcoRI-induced DSBs in screens utilizing both *MATa* and *MATα* deletion strain libraries. Most mutants were also sensitive to the clastogenic chemicals MMS and bleomycin. Several of the non-RAD52 group genes have previously been linked to DNA repair and over half of the genes affect nuclear processes. Many proteins encoded by the protective genes have previously been shown to associate physically with each other and with known DNA repair proteins in high-throughput proteomics studies. A majority of the proteins (64%) share sequence similarity with human proteins, suggesting that they serve similar functions.

**Conclusions:**

We have used a genetic screening approach to detect new genes required for efficient repair of DSBs in *Saccharomyces cerevisiae*. The findings have spotlighted new genes that are critical for maintenance of genome integrity and are therefore of greatest concern for their potential impact when the corresponding gene orthologs and homologs are inactivated or polymorphic in human cells.

## Background

Chromosomal DNA within eukaryotic cells is constantly subjected to many types of damage from both endogenous and exogenous sources. Repair of the damage may be performed by one or more different pathways, depending on the specific type of DNA lesion involved. Major pathways that have been characterized include nucleotide excision repair (NER), active on ultraviolet light induced DNA crosslinks and other bulky lesions, base excision repair (BER) for repair of damaged and lost bases, and mismatch repair (MMR). Two other pathways, homologous recombination (HR) and nonhomologous end-joining (NHEJ), are active in repair of DNA double-strand breaks (DSBs). DSBs can be induced by endogenous processes such as oxidation, arrest of DNA replication forks, nuclease cleavage or processing of various DNA lesions and by exposure to external agents such as ionizing radiation or clastogenic chemicals [1,2]. DSB ends may be blunt or may contain single-strand overhangs. The ends may also have other associated damage such as altered or missing bases, e.g., as seen after cells are exposed to ionizing radiation or the chemical bleomycin [1-4]. Most DNA damaging agents that induce DSBs generate other lesions as well, with the other lesions typically occurring at much higher frequencies. Despite their rarity, unrepaired and/or misrepaired DSBs are believed to be the major cause of cell death after exposure to such agents [5,6].

Repair of DSBs by NHEJ and HR involves the concerted actions of many conserved proteins found in both single-celled and multicellular organisms. In *Saccharomyces cerevisiae*, or budding yeast, NHEJ requires at least three factors. The Yku70/Yku80 complex binds to DNA ends, provides protection from nucleases, and recruits other repair proteins. The Mre11/Rad50/Xrs2 (Mrx) complex forms a bridge between the broken DNA ends and also has nuclease activities involved in processing of some end structures. Dnl4/Lif1/Nej1 represents the DNA Ligase IV complex, which covalently links broken DNA strands together [7-10]. Nej1 may also function earlier in the pathway, modulating the binding of Yku70/Yku80 to DNA ends [11]. The chromatin silencing proteins Sir2, Sir3 and Sir4 are also required in yeast cells, but they primarily affect NHEJ indirectly through their effects on *NEJ1* gene expression [7]. Other proteins such as Exo1, Rad27, Pol4, the Rsc and Smc complexes, and DNA damage checkpoint proteins have also been suggested to modulate the efficiency of NHEJ repair [7,8,10,12,13].

Repair by the homologous recombination pathway involves association of broken DSB ends with an unbroken homologous DNA molecule such as a sister chromatid or a homologous chromosome, which then serves as a template for repair. Efficient repair by this pathway requires the functions of many proteins, especially several members of the RAD52 group (Rad50, Rad51, Rad52, Rad54, Rad55, Rad57, Rad59, Mre11, Xrs2 and Rdh54) [14-17]. This group of genes was originally defined in the 1970s on the basis of mutant phenotypes. With the exceptions of *RAD59* and *RDH54*, which are weak homologues of *RAD52* and *RAD54*, respectively, mutants with defects in these genes share many common phenotypes. They exhibit strong sensitivity to ionizing radiation and chemical clastogens, weak sensitivity to ultraviolet light, and genetic epistasis when combined with mutations in other members of the group to generate double mutants [18]. Two other original members of this group are *RAD53*, now known to function primarily in the DNA damage-induced checkpoint response, and *RAD56*, a locus that remains uncharacterized [18]. An additional gene, *RAD61*, has also been suggested as a member, though its main function may lie in sister chromatid cohesion [19,20].

The general model for recombinational repair of DSBs involves several steps. First, broken ends are resected by Mrx and other enzymes such as Exo1, Sgs1, Sae2 and Dna2 to create long 3’-ended single-stranded DNA (ssDNA) tails. The tails are subsequently bound by the ssDNA binding protein complex (Rpa), Rad51, and other proteins that recruit factors promoting a homology search, strand invasion of an unbroken DNA molecule, new DNA synthesis and other steps ultimately leading to repair of the broken strands and separation (resolution) of the paired complexes [14-17]. Several additional proteins have been shown to influence the efficiency of the pathway. These include components of the DNA damage checkpoint response (e.g., Mec1, Mec3, Rad9, Rad17, Rad24, etc.), nucleosome remodeling (the Rsc complex), sister chromatid cohesion (Eco1, Smc5, Smc6, Scc2, etc.), other end-processing nucleases (Rad1/Rad10, Msh2/Msh3, Saw1, etc.), resolvase-like enzymes (Mus81/Mms4, Slx1/Slx4, Yen1, etc.) and chromatin reassembly proteins [21-26].

The development of yeast deletion strain libraries has allowed researchers to screen thousands of individual gene mutants in a single experiment to identify all mutants that have a particular phenotype. Several previous studies have involved testing such strains for sensitivities to physical or chemical agents that damage DNA, including ionizing radiation, ultraviolet light, methyl methanesulfonate (MMS) and other chemicals [19,27-31]. Genomic screens by Bennett *et al.* and Game *et al.* using over 4700 diploid mutants identified a large number of genes that are required for resistance to gamma radiation [19,27,28,32]. Ionizing radiation generates low levels of DSBs in cellular DNA, but it induces higher frequencies of many other types of DNA damage and also causes damage to other macromolecules inside the cell. Thus, only a subset of the genes found to be required for radiation resistance are actually likely to affect DSB repair.

The bacterial restriction endonuclease EcoRI recognizes and cuts the palindromic 6 bp sequence G^AATTC, generating 5’ ssDNA overhangs that are 4 nt long. Controlled *in vivo* expression of this nuclease from a galactose-regulated promoter has been employed in several studies investigating cellular responses to DSBs and their repair [33-36]. Use of EcoRI was advantageous for these studies because it is believed to produce essentially only one type of DNA lesion, a DSB. Thus, it is more specific than commonly used clastogens like radiation, MMS or bleomycin. EcoRI-induced DSBs are repaired efficiently in most wildtype haploid strains of yeast, but produce strong growth inhibition and modest killing in most recombination-deficient RAD52 group mutants [34,37]. In contrast, the impact of EcoRI expression on NHEJ mutants (e.g., *yku70* or *dnl4* cells) is more variable and dependent on the strain background employed for the study. This characteristic is similar to the variable sensitivities to MMS and bleomycin that have been observed in NHEJ mutants [6,38].

Progress in understanding DSB repair pathways has been hindered because many of the proteins acting on or influencing efficiency at each of the various steps have remained unidentified. In the current study we tested each of the genes shown previously to be important for resistance to ionizing radiation, using both *MATα* and *MATa* haploid mutant libraries, to identify those genes that specifically affect DSB repair. This goal was accomplished by screening the mutants for sensitivity to DSBs produced by *in vivo* expression of EcoRI. Use of two libraries was advantageous because it allowed phenotypes observed in one haploid mutant to be confirmed in an equivalent mutant of opposite mating type. These efforts have resulted in the identification and characterization of 73 non-RAD52 group genes that are required for efficient repair of site-specific DSBs.

## Results

Two previous screens undertaken using diploid yeast deletion strain libraries (collections of > 4700 yeast strains, each with both copies of a specific gene knocked out), identified a total of 210 mutants with reduced resistance to gamma radiation [19,27,28,32]. Several of these mutants corresponded to known RAD52 group genes defective in recombination, but most genes had not previously been associated with DNA repair. Because radiation generates many types of cellular damage, only a subset of these genes are likely to affect DSB repair. To identify genes specifically impacting repair of DSBs, haploid *MATα* versions of each of the 210 mutants were tested for resistance to DSBs produced by *in vivo* expression of EcoRI endonuclease from a *GAL1* promoter. One more library mutant, *exo1*, was added to the screen, bringing the total to 211. Exo1 is a 5’-to’3’ exonuclease active in resection of DSB ends in the homologous recombination pathway and it has other roles in replication and mismatch repair [17,39]. Although *exo1*^*−/−*^ cells were not detected in the original diploid screens, we have previously observed that *exo1* haploid library mutants are gamma-sensitive (described below) and human *exo1*^*−/−*^ cells are sensitive to ionizing radiation [40].

**Figure 1:**
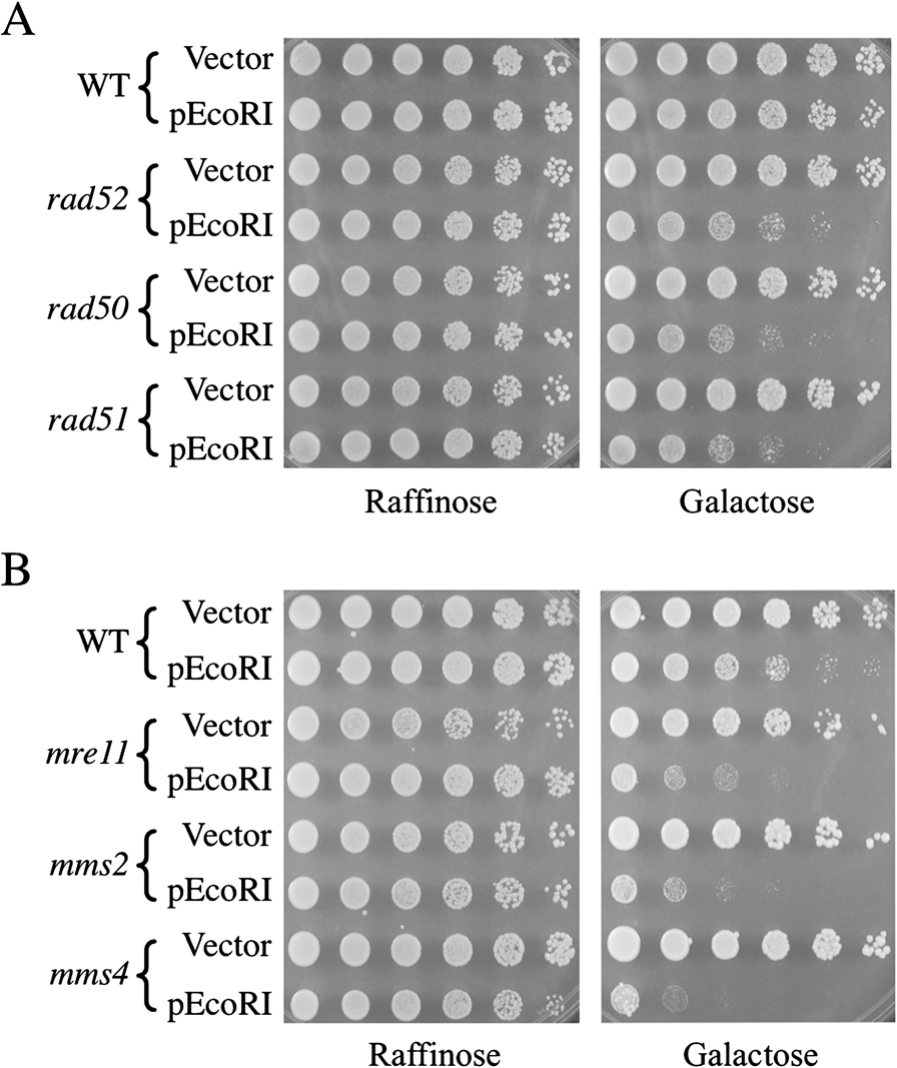
**Survival of control strains and new haploid DSB repair mutants when EcoRI is expressed *in vivo *from a galactose-inducible promoter.** (**A**) Colony forming ability and cell growth rate are reduced in recombination-deficient *rad50, rad51* and *rad52* mutants. (**B**) Example of pronging plate assay used to screen *MATα* library mutants for EcoRI sensitivity. Cells contained either vector (pRS316) or YCpGal::RIb. Cells grown in raffinose media were serially diluted 5-fold and pronged onto plates containing either raffinose or galactose.

For these experiments, growth of cells containing the control vector pRS316 or the *GAL1p::EcoRI* fusion plasmid YcpGal::RIb [33,34] was initially analyzed by “double-imprint” replica-plating patches of cells from plates containing 2% raffinose to plates containing 2% galactose (see Methods). Mutants whose growth was inhibited in the presence of galactose were then retested using more quantitative dilution pronging survival assays. For each pronging assay, cells containing either the vector or the *GAL1p::EcoRI* plasmid were harvested from patches on raffinose plates, serially diluted 5-fold into 96-well microtiter dishes, and pronged to plates containing raffinose or galactose. Tests of control *MATα* haploid library strains revealed that survival of wildtype BY4742 cells expressing EcoRI was high. In contrast, cell survival (determined by colony numbers) and growth rates (indicated by colony diameters) were reduced in several RAD52 group recombination mutants (*rad50*, *rad51*, *rad52*, etc.) (Figure 1A). Colony formation in mutants defective in NHEJ repair (*yku70*, *yku80, sir2, sir3*, *dnl4*, *nej1*, etc.) was similar to that of wildtype cells in this strain (BY4742 cells, S288c background) (data not shown). This phenotype was not unexpected because DNA damage sensitivities of NHEJ mutants are known to vary in different strain backgrounds [6,38].

Testing of the 211 haploid *MATα* library mutants revealed that survival of many of the strains was reduced by expression of EcoRI. Results obtained with two mutants, *mms2* and *mms4*, are depicted in Figure 1B along with WT and *mre11* strain controls. Interestingly, 187 of the 211 mutants were successfully evaluated using the procedure described above, but 24 of the *MATα* library strains could not be tested, usually because they grew too poorly on galactose plates. Some mutants could not be tested for other reasons. *ade12* mutants did not produce Ura^+^ colonies even after repeated transformations with pRS316 and YCpGal:RIb plasmid DNAs. Also, *cdc40* cells from the library were phenotypically Ura^+^, preventing use of the *URA3* selectable marker on the two plasmids. A total of 62 of the 187 *MATα* mutants that could be tested exhibited killing when EcoRI was expressed.

To address the 24 *MATα* library strains that were untestable, 24 haploid mutants with the equivalent genes inactivated were obtained from an alternative deletion strain collection of opposite mating type. Twenty-two of these 24 *MATa* strains grew on galactose media and could be successfully transformed with the assay plasmids. This result suggests that many of the 24 original *MATα* library versions of the strains had secondary mutations affecting carbohydrate metabolism. Only two of the *MATa* mutants, *ade12* and *hfi1*, remained untestable. Similar to the *MATα* library strains, transformations of *MATa ade12* mutants did not produce Ura^+^ colonies, and *hfi1* cells from both libraries grew too poorly to be tested.

Eleven of the 22 *MATa* mutants were found to be EcoRI-sensitive (EcoRI^s^). Thus, 209 of the original 211 genes were tested, 187 as *MATα* strains and 22 as *MATa* cells, and 81 of them were identified as important for normal resistance to EcoRI-induced DSBs in haploid cells. As shown in the top section of Table 1, eight members of the RAD52 group (*rad50, rad51, rad52, rad54, rad55, rad57, mre11* and *xrs2*) were required for normal resistance to EcoRI. Deletions of two other group members, *RAD59* and *RDH54*, had no detectable effect. Another gene proposed to be a member of the group called *RAD61*[19] was also not required for repair of the EcoRI-induced DSBs. Both *MATa* and *MATα* versions of *rad59*, *rdh54* and *rad61* strains were tested and found to be resistant to EcoRI (data not shown). All mutants in Table 1 were classified as moderately sensitive (S) or strongly sensitive (SS), based on the extent of colony formation seen in the semi-quantitative pronging survival assays (described in Methods).

**Table 1:** **Library mutants identified as EcoRI-sensitive in *MATα *cells*, MATα *cells*, *or both *MATα *and *MATα *strains**^**a,b**^

**Mutant**	***MATα***	***MATa***	**Mutant**	***MATα***	***MATa***
*mre11*^*c*^	S	S	*rad54*	S	S
*rad50*	S	S	*rad55*	S	S
*rad51*	S	S	*rad57*	S	R
*rad52*	S	S	*xrs2*	N/D	S
*adk1*	S	S	*mrps35*	S	N/D
*ado1*	S	S	*not5*	SS	SS
*akr1*	SS	S	*nup84*	S	SS
*apq13/net1*	SS	S	*och1*	S	N/D
*arp5*	N/D	S	*psy1/ykl075c*	S	R
*atp2*	SS	S	*rad5*	S	R
*bck1*	SS	S	*rtt109(rem50)*^*d*^	S	S
*bik1*	N/D	S	*rpb9*	S	SS
*bud19/rpl39*	SS	SS	*rpl31a*	S	S
*bud30/rpc53*	N/D	SS	*rsm7/yjr114w*	N/D	S
*bud32*	N/D	S	*rtf1*	N/D	SS
*bur2*	S	SS	*rvs161*	S	SS
*cax4*	SS	SS	*sae2*	S	S
*ccr4*	N/D	S	*sam37*	SS	SS
*cdc40*	N/D	SS	*sco1*	N/D	SS
*cgi121*	SS	SS	*sfp1*	SS	S
*cis3*	SS	S	*slm4*	S	S
*cnm67*	SS	S	*spt10*	S	S
*ctf4*	SS	SS	*spt20*	S	R
*ctf8*	SS	S	*srv2*	S	S
*dcc1*	SS	SS	*taf14*	SS	S
*ddc1*	S	S	*trm9*	S	SS
*eaf1/opi7*	S	N/D	*tsr2/ylr434c*	S	S
*exo1*	S	S	*ubp8*	S	SS
*gcn5*	SS	S	*ubr1*	S	N/D
*gnd1/yhr182c-a*	SS	SS	*ume6*	SS	S
*hsp150*	SS	S	*vma7*	SS	S
*htl1*	SS	SS	*vph2/ykl118w*	SS	S
*ids2*	S	SS	*ybr099c/mms4*	SS	S
*img2*	SS	N/D	*ydr417c/rpl12b*	S	N/D
*lip5*	S	S	*ydr433w/npl3*	SS	N/D
*lrp1*	SS	S	*ygl218w/mdm34*	N/D	SS
*lsm7*	S	SS	*ylr235c/top3*	S	S
*mct1*	S	SS	*yml009w-b/spt5*^*e*^	SS	S
*mms2*	S	S	*yml012c-a/ubx2*	S	S
*mms22*	SS	SS	*ynr068c*	S	S
*mms4/ybr099c*	SS	SS			

^a^ Eight RAD52 group mutants are shown at the top of the table. Mutants were ranked as resistant (R), sensitive (S), or strongly sensitive (SS) (see Methods). N/D usually indicates that the library strain was unable to grow on galactose plates. Some genes could not be tested for other reasons, e.g., *MATα cdc40* cells were Ura^+^.

^b^ Gene names separated by a forward slash indicate deletions affecting two or more overlapping open reading frames. The coding region of the gene listed first was deleted in each strain.

^c^ The *MATα mre11* strain was reconstructed for this study because the library mutant did not exhibit phenotypes characteristic of *mre11* mutants (MMS- and gamma-sensitivity). ^d^*RTT109* is usually referred to as *REM50* in the literature. ^e^*yml009w-b* overlaps the verified gene *spt5* and another open reading frame called *yml009c-a.*

Because of the apparent high frequency of secondary mutations in the original library, experiments were performed to determine if EcoRI sensitivities seen in the *MATα* mutants could be confirmed in *MATa* versions of the strains. The EcoRI^s^ phenotype was reproduced in 59 of the 62 equivalent *MATa* strains using dilution pronging as before, with only *psy1*, *rad5* and *spt20 MATa* library strains exhibiting resistance (Table 1). These experiments therefore identified 8 RAD52 group mutants and 73 non-RAD52 group mutants as important for survival after induction of EcoRI. For 59 of the latter mutants, sensitivity was confirmed in both *MATα* and *MATa* strains. For completeness, all of the library mutants that were tested in this study and found to be resistant to EcoRI are listed in Additional file 1: Table S1.

**Figure 2:**
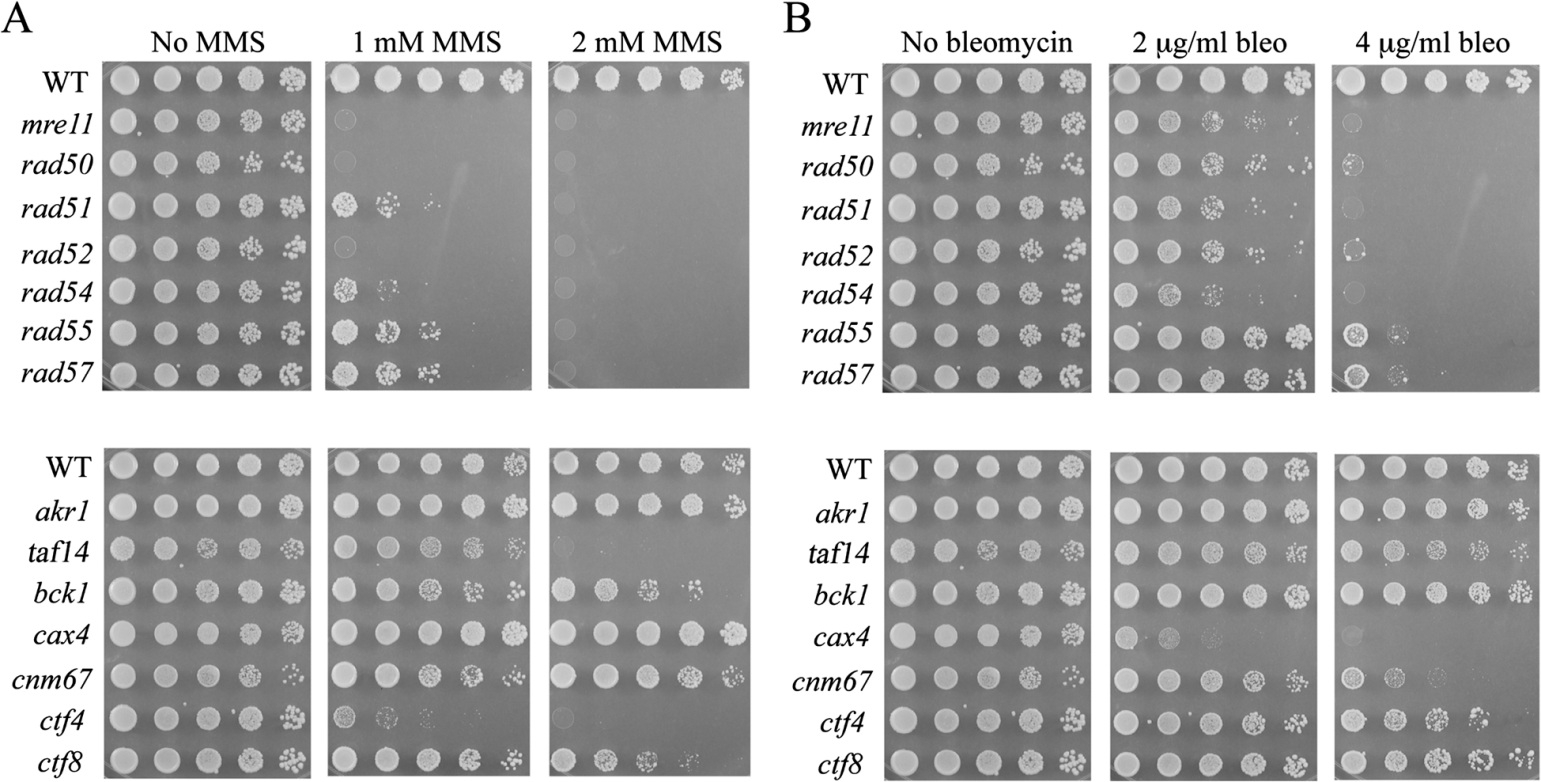
**Survival assays used for determination of sensitivities to chemical clastogens.** (**A**) MMS, (**B**) bleomycin. Plates contained control recombination-defective RAD52 group mutants (top panels) or deletion library strains (bottom panels).

The 81 mutants exhibiting sensitivity to EcoRI were further characterized by assessing their survival after exposure to the chemicals MMS and bleomycin. These chemicals induce DSBs by very different mechanisms and have been widely used to investigate DNA repair pathways [3,4,41]. The eight RAD52 group mutants plus all 73 other mutants were analyzed using dilution pronging survival assays. Strains from the original *MATα* library were used for the experiments, with *MATa* library strains substituted only for *MATα* cells that had growth defects. RAD52 group mutants exhibited strong killing on plates containing either 2 mM MMS or 4 μg/ml bleomycin (Figure 2A and B, top panels; 7 of the 8 RAD52 group mutants are shown in this representative figure). *rad55* and *rad57* mutants were least sensitive in the assays, which were conducted at 30°C; these mutants exhibit greatest sensitivity to DNA damaging agents at 23°C [18]. Higher concentrations of MMS and bleomycin were not used because they caused strong growth inhibition of WT cells (not shown). Many of the 73 non-RAD52 group mutants were also sensitive to killing by MMS or bleomycin (e.g., Figure 2A and B, bottom panels). Results of all survival tests are summarized in Table 2. In total, 53 of the 73 EcoRI^s^ non-RAD52 group mutants were sensitive to bleomycin (4 μg/ml) and 44 mutants were sensitive to MMS (2 mM). Surprisingly, growth of 9 strains was not affected by either MMS or bleomycin. The pronging assays are limited to detection of mutants that consistently exhibit ≥ 2 fold fewer colonies than wildtype cells after exposure to DNA damage. It is possible that modest chemical sensitivities in some mutants could not be detected by this method.

**Table 2:** **Survival of EcoR1**^**s **^**mutants after exposure to MMS, bleomycin or gamma radiation**^**a**^

**Mutant**	**MMS (mM)**		**bleo (μg/ml)**		**Γ (krads)**		**Mutant**	**MMS (mM)**		**bleo (μg/ml)**		**Γ (krads)**	
	**1**	**2**	**2**	**4**	**30**	**60**		**1**	**2**	**2**	**4**	**30**	**60**
RAD52 group:													
*rad50*	SS	SS	SS	SS	SS	SS	*rad55*	SS	SS	R	SS	SS	SS
*rad51*	SS	SS	SS	SS	SS	SS	*rad57*	SS	SS	R	SS	SS	SS
*rad52*	SS	SS	SS	SS	SS	SS	*mre11*	SS	SS	SS	SS	SS	SS
*rad54*	SS	SS	SS	SS	SS	SS	*xrs2*	SS	SS	SS	SS	SS	SS
New EcoRI^s^ mutants:													
*adk1*	R	R	SS	SS	R	R	*mrps35*	R	S	R	S	R	S
*ado1*	S	S	SS	SS	S	S	*not5*	R	R	R	R	R	R
*akr1*	R	R	R	R	R	S	*nup8*	R	S	S	SS	R	R
*apq13*	S	SS	S	SS	R	R	*och1*	R	S	S	SS	R	R
*arp5*	R	S	R	R	R	S	*psy1*	R	R	R	R	R	R
*atp2*	S	SS	S	S	R	R	*rad5*	SS	SS	S	SS	R	R
*bck1*	S	SS	R	R	R	S	*rem50*	R	SS	R	S	R	R
*bik1*	R	R	R	R	R	R	*rpb9*	R	SS	S	SS	S	SS
*bud19*	S	S	SS	SS	S	SS	*rpl31a*	R	R	R	R	R	R
*bud30*	R	R	S	S	S	S	*rsm7*	R	R	S	SS	R	R
*bud32*	R	R	SS	SS	S	SS	*rtf1*	R	S	R	SS	S	SS
*bur2*	R	S	R	R	R	R	*rvs161*	R	R	R	SS	R	S
*cax4*	R	R	SS	SS	S	SS	*sae2*	R	S	R	S	R	S
*ccr4*	R	R	S	SS	R	R	*sam37*	S	SS	S	SS	R	R
*cdc4*	SS	SS	SS	SS	SS	SS	*sco1*	R	R	R	R	R	R
*cgi121*	R	R	S	S	SS	SS	*sfp1*	R	R	R	S	R	R
*cis3*	S	SS	R	S	R	R	*slm4*	S	SS	S	SS	R	R
*cnm67*	R	S	R	SS	S	SS	*spt10*	R	S	SS	SS	SS	SS
*ctf4*	SS	SS	R	S	R	R	*spt20*	R	R	S	SS	R	R
*ctf8*	S	SS	R	S	R	S	*srv2*	R	R	R	R	R	R
*dcc1*	S	SS	R	S	R	R	*taf14*	S	SS	R	S	R	R
*ddc1*	R	SS	R	R	R	R	*trm9*	S	S	S	S	R	R
*eaf1*	R	S	R	S	S	S	*tsr2*	R	R	R	S	R	S
*exo1*	R	R	R	R	S	S	*ubp8*	R	R	R	R	R	R
*gcn5*	R	SS	R	S	R	R	*ubr1*	S	S	R	S	SS	SS
*gnd1*	S	S	R	SS	S	SS	*ume6*	R	R	R	R	R	S
*hsp150*	R	R	R	R	R	R	*vma*	S	S	SS	SS	S	SS
*htl1*	R	S	SS	SS	SS	SS	*vph2*	R	R	SS	SS	R	R
*ids2*	R	R	R	R	R	S	*ybr099c*	S	SS	R	R	R	R
*img2*	R	R	R	S	R	R	*ydr417c*	S	SS	S	SS	R	R
*lip5*	R	R	R	S	S	SS	*ydr433w*	R	SS	SS	SS	R	R
*lrp1*	R	R	R	S	R	R	*ygl218w*	R	R	R	R	R	R
*lsm7*	S	S	R	S	R	S	*ylr235c*	SS	SS	R	S	R	S
*mct1*	S	SS	R	S	S	SS	*yml009w-b*	S	SS	R	S	R	R
*mms2*	S	SS	R	R	R	R	*yml012c-a*	S	SS	R	S	R	R
*mms22*	SS	SS	R	S	S	S	*ynr068c*	SS	SS	S	SS	R	R
*mms4*	S	SS	R	R	R	R							

^a^ Mutants were ranked as resistant (R), sensitive (S), or strongly sensitive (SS). Mutants classified as SS exhibited > 25-fold higher killing than wildtype cells using semi-quantitative dilution pronging assays. Γ, gamma radiation.

**Figure 3:**
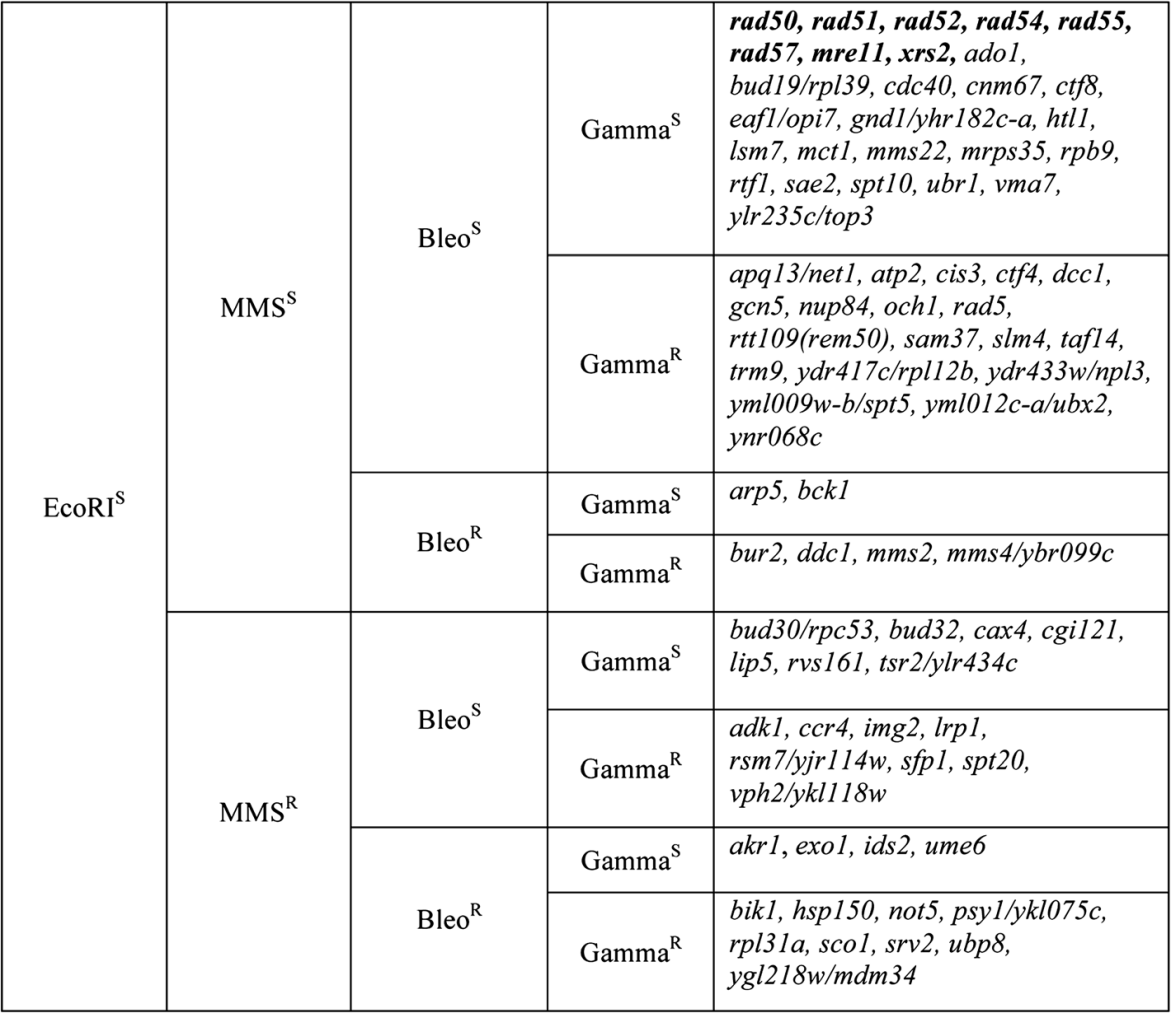
**Classification of 81 EcoRI**^**s **^**haploid library mutants based on sensitivities to MMS, bleomycin and ionizing radiation.** RAD52 group mutants are shown in boldface.

The results described above established that all 8 EcoRI^s^ RAD52 group mutants were sensitive to MMS and bleomycin and that most of the 73 other EcoRI^s^ mutants were also sensitive to one or both of the chemical clastogens. Survival of each of the mutants after exposure to a single dose of gamma radiation (30 or 60 krads) was tested next using dilution pronging assays. Each of the RAD52 group mutants was strongly sensitive to ionizing radiation but, unexpectedly, only 32 of the other 73 mutants showed cell killing at 60 krads (Table 2). Each of these genes had been shown previously to affect radiation resistance in diploids [19,27,28,32], but over half of them had no discernible impact on survival in the current haploid assays. Clastogen sensitivities for all 81 mutants are depicted schematically in Figure 3. The recombination-deficient RAD52 group mutants (shown in boldface) were sensitive to all 4 DSB-inducing agents, but only 19 of the remaining 73 mutants were similarly sensitive to all agents (Figure 3, upper right panel). The genes inactivated in these 19 strains therefore affect sensitivity to enzymatic, chemical and physical DSB-generating agents, suggesting that they are most likely to affect DSB repair processes directly.

The 73 non-RAD52 group mutants showed reduced survival when EcoRI was expressed and most of the strains were also sensitive to MMS and bleomycin, but only 32 were killed by radiation. Differences in the methodologies employed for the assays may have contributed to this discrepancy. In the current study, radiation resistance was determined after a single exposure of cells to gamma radiation lasting less than 60 min. The cells were then incubated at 30°C on agar plates for several days, without further exposure, until the surviving cells grew into colonies and could be counted. In the EcoRI, MMS, and bleomycin experiments, however, cells were exposed continuously to these clastogens as they grew on agar plates for a similar number of days (MMS and bleomycin were incorporated into the agar and EcoRI was expressed continuously from the *GAL1* promoter). Unlike the irradiated cells, these cells traversed multiple cell cycles while continuously exposed to DSBs. Cells are particularly sensitive to some types of DNA damage in specific phases of the cell cycle, especially during S phase and mitosis [42,43]. The different result obtained with radiation suggested the possibility that some genes were needed for survival when damage was constantly generated, but not in cells that experienced damage only one time in a single phase of the cycle.

Bleomycin damages DNA directly by entering cells, forming a free radical complex, and binding and cleaving DNA in the nucleus. Cells can be exposed to this clastogen continuously by adding it to liquid or plate cell growth media. It is also possible to perform a single exposure experiment by adding bleomycin to cells for a brief time, e.g., 30 min, and then washing the cells several times to remove the drug before spreading onto plates to determine survival. Most of the new mutants identified here were killed by continuous exposure to bleomycin in plates, but not by a single brief exposure to gamma radiation, which also generates DSBs primarily by free radical mechanisms [1-3]. We hypothesized that these mutants would be similarly resistant to a brief exposure to bleomycin if continuous exposure was the key to their sensitivity.

To assess survival after a single exposure to bleomycin, cells were harvested from patches on plates, inoculated into YPDA broth and grown to log phase at 30°C, followed by exposure to 10 μg/mL bleomycin for 30 min (similar to the protocol used for the gamma radiation survival experiments). Cells were washed, 10-fold serial dilutions were made and appropriate volumes of the diluted cultures were spread onto YPDA plates and incubated at 30°C for three to four days to detect surviving colonies. Survival was strongly reduced in control *rad52* cells and moderately reduced in *rad57* mutants, which is in accord with their previously characterized phenotypes as part of the RAD52 group (Table 3). Five non-RAD52 group mutants that were resistant to a brief exposure to gamma (*ctf4*, *nup84, rem50, slm4* and *taf14*) were tested for their sensitivities to brief treatment with bleomycin. Survival of the gamma-resistant mutants was variable, ranging from near-wildtype in *taf14* mutants to a modest 100-fold reduction in *ctf4* mutants (similar to *rad57* cells). We then reasoned that there might be a different correlation: the gamma resistant mutants may simply exhibit a range of bleomycin sensitivities that is different than the range of sensitivities found among gamma sensitive mutants. However, the five gamma sensitive mutants tested (*cnm67*, *htl1*, *mms22*, *rpb9* and *ubr1*) showed sensitivities that were similar to the gamma resistant strains, with survival reduced from 8.1 fold to 43.5 fold (Table 3, bottom rows). The median fold reduction for the gamma sensitive mutants was only moderately larger than that of the gamma resistant strains (19.6-fold versus 7.6-fold). These experiments indicate that there is not a simple correlation between sensitivities to a single brief exposure to bleomycin and to gamma radiation. They also suggest that the small number of gamma sensitive mutants relative to EcoRI, MMS and bleomycin sensitive mutants is unlikely to be due simply to different treatment methods involving continuous versus single-hit exposures.

**Table 3:** Survival of haploid yeast cells after a single brief exposure to the antitumor drug bleomycin

**Strain**	**Gamma**	**Bleomycin**	**Fold reduction**
	**(60 krad)**	**(10 ug/ml)**	
WT	R	100 ± 10.7%	
*rad57*	SS	0.8 ± 0.3%	125×
*rad52*	SS	0.04 ± 0.01%	2500×
*taf14*	R	96.6 ± 28.3%	1.0×
*slm4*	R	62.8 ± 9.3%	1.6×
*nup84*	R	13.2 ± 1.2%	7.6×
*rem50*	R	8.4 ± 2.5%	11.9×
*ctf4*	R	1.0 ± 0.7	100×
*cnm67*	S	12.3 ± 2.9%	8.1×
*rpb9*	SS	6.3 ± 1.1%	15.9×
*mms22*	S	5.1 ± 2.0%	19.6×
*htl1*	SS	2.9 ± 0.3%	34.5×
*ubr1*	SS	2.3 ± 1.0%	43.5×

Averages of four trials and standard deviations are shown.

**Figure 4:**
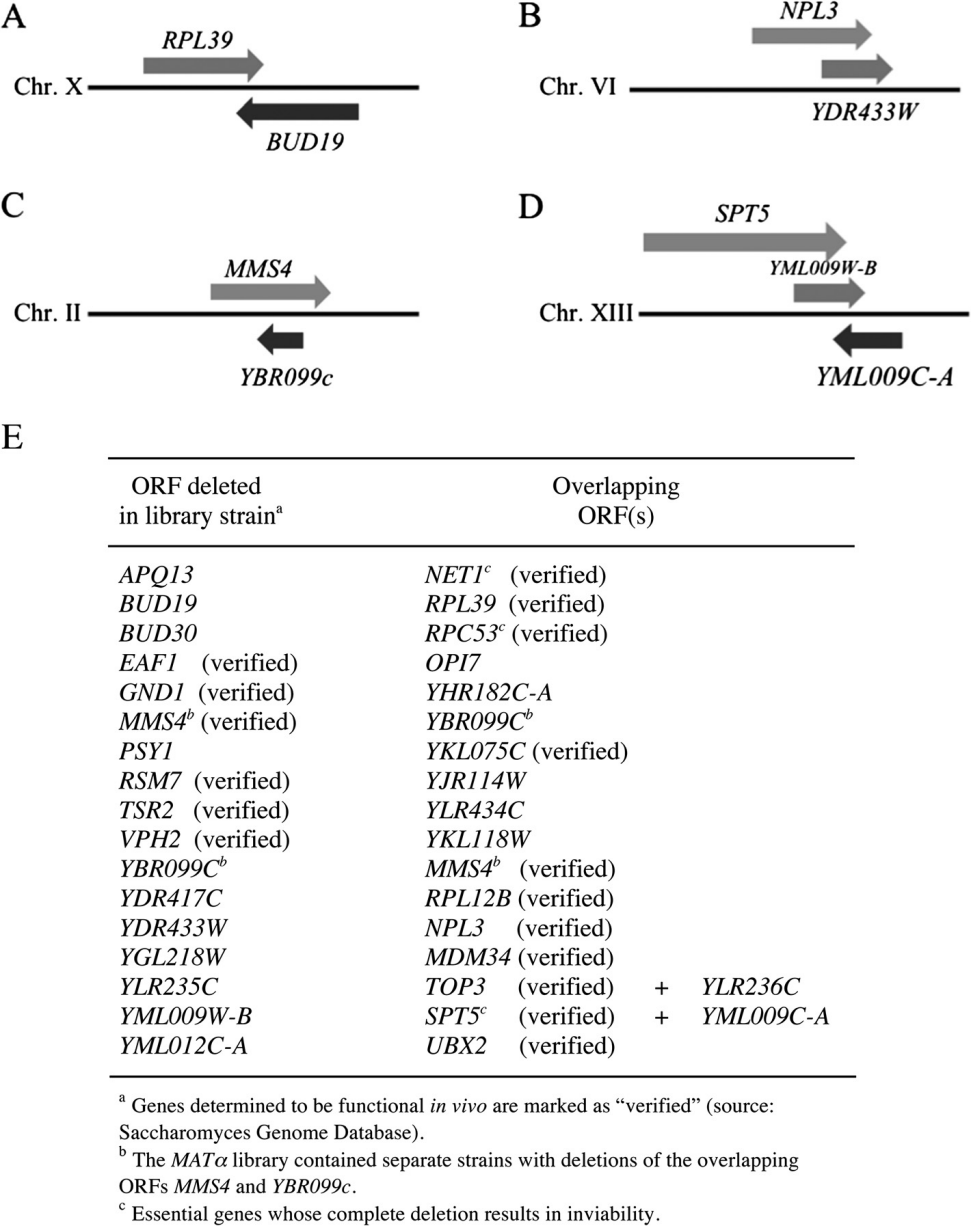
**Many library mutants contain a deletion of an open reading frame (ORF) that also deletes part or all of an overlapping ORF.** (**A-D**) Illustrations depicting overlapping genes found at four chromosomal loci. (**E**) ORFs whose coding regions were precisely deleted in the library strains and overlapping ORFs affected by the deletion. Genes listed as “verified” are known to produce a protein product (source: Saccharomyces Genome Database). All other ORFs are unverified.

The Saccharomyces Genome Database (http://www.yeastgenome.org) contains detailed descriptions of all known and putative genes within the *Saccharomyces cerevisiae* genome. This resource was utilized to investigate the functions and potential relationships among the genes affecting survival after induction of EcoRI expression. Analysis of the chromosomal locations of each of the genes revealed that overlapping of two putative open reading frames (ORFs) was a characteristic of 16 of the loci. When overlapping occurs, it is not completely certain that loss of the gene that was precisely deleted in the library mutant (from its start codon to its stop codon) has caused the observed phenotype. Examples are shown in Figure 4 (A – D), illustrating four different orientations of overlapping genes. Most of the overlapping ORFs are predicted to be transcribed in different directions, but in some cases each ORF was oriented in the same direction. In most cases, one of the ORFs has been shown experimentally to produce a protein *in vivo*, but transcription/translation of the other putative gene has not yet been established. ORFs that were specifically deleted in the library strains used for this project are shown in Figure 4E (left column) along with the overlapping ORFs that were partially deleted (right column). At only one locus, containing *MMS4* and *YBR099C*, were both ORFs separately deleted during the construction of the haploid strain library. Thus, both of these genes are listed in the left column of Figure 4E.

**Figure 5:**
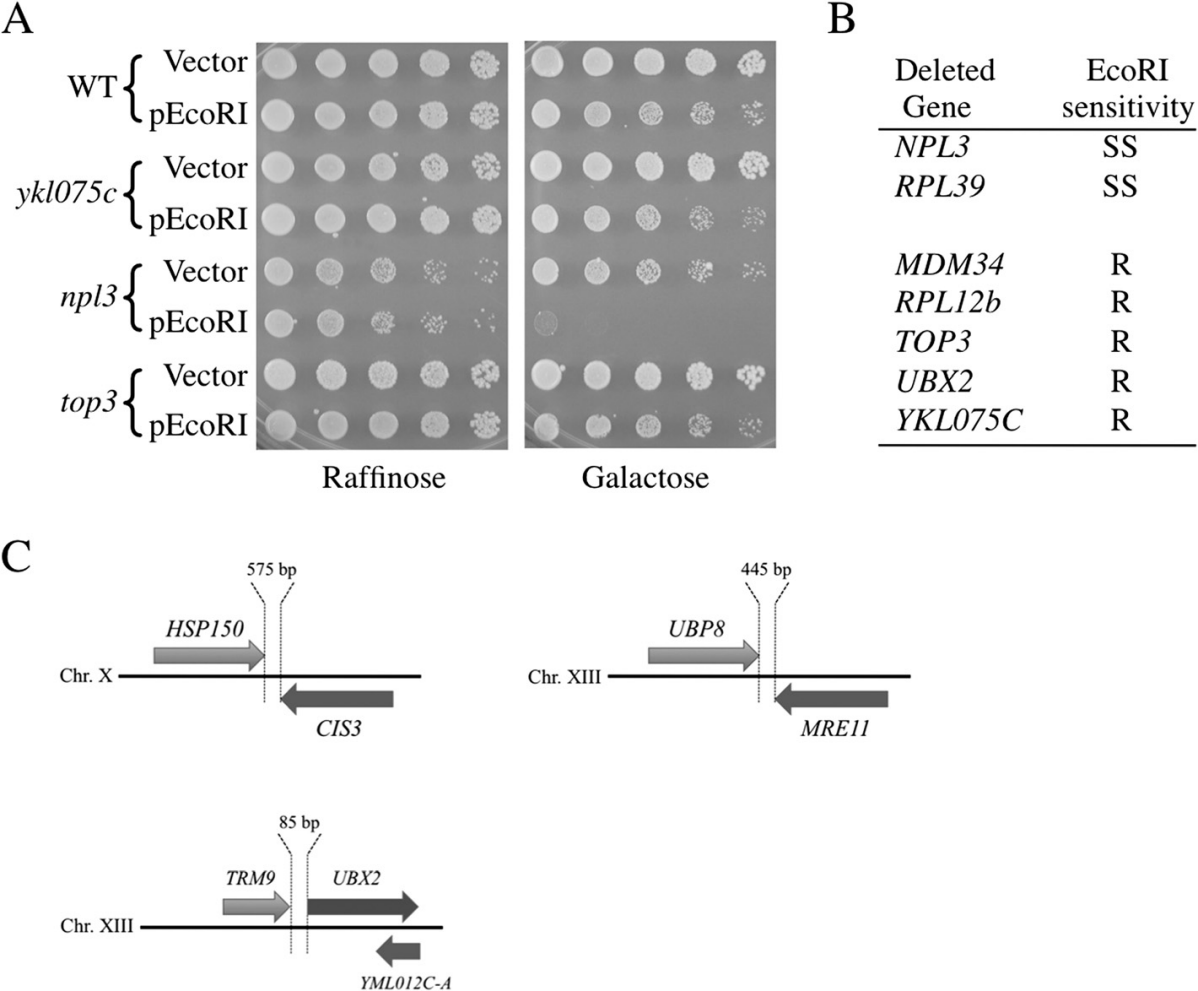
**Analysis of overlapping and adjacent ORFs affecting EcoRI sensitivity.** (**A**) Example survival assay performed to determine if inactivation of three verified genes (*YKL075C, NPL3*, and *TOP3*) that overlap ORFs required for EcoRI resistance also affect resistance to EcoRI. (**B**) Deletion of two of the seven overlapping, verified genes resulted in strong killing by EcoRI. SS, strongly sensitive; R, resistant. (**C**) Schematic representations of loci containing two genes required for EcoRI resistance that are adjacent to each other.

At six of the loci, the gene that was precisely deleted has previously been shown to produce a protein product (indicated as “verified”). At most of the loci the ORF that was deleted is unconfirmed and overlaps a verified gene. This suggests that some mutants analyzed here (and in previous mutant library screens) may exhibit phenotypes because of partial deletions of overlapping verified genes, not because of the ORF whose coding region was deleted. To investigate this possibility, *MATα* library mutants containing precise deletions of the coding regions of 7 of the verified overlapping genes (*RPL39, YKL075C, RPL12B, NPL3, MDM34, TOP3* and *UBX2*; Figure 4E) were tested for sensitivity to EcoRI. Two of the strains, *npl3* and *rpl39*, were strongly sensitive to EcoRI expression (Figure 5A and B), suggesting that inactivation/truncation of these verified genes may have been the actual cause of the EcoRI^s^ phenotype in the original mutants. Growth of the remaining 5 mutants was not affected by EcoRI expression. The source of the sensitivities of the original mutants with these latter 5 genes inactivated remains unclear. It may be due to inactivation of the unverified genes (whose transcription has not yet been confirmed) or it may be caused by production of truncated proteins from the partially deleted verified genes that produce phenotypes not seen when the genes are completely inactivated.

Surprisingly, some of the 81 genes required for resistance to EcoRI were found to lie directly adjacent to each other. When two genes are adjacent, deletion of one gene has the potential to exert polar effects on the transcription and/or mRNA stability of the nearby gene. At two loci on chromosomes X and XIII, the *HSP150* and *CIS3* genes and the *UBP8* and *MRE11* genes were found to be only 575 bp and 445 bp from each other, respectively (Figure 5C). At another region on chromosome XIII, the coding regions of *TRM9* and *UBX2* are remarkably close (separated by only 85 bp), with the intergenic segment encompassing the entire presumptive promoter region of *UBX2* (Figure 5C).

The Locus Summary gene descriptions and Gene Ontology (GO) annotations indicating Biological Processes compiled at the Saccharomyces Genome Database were employed to analyze and sort the non-RAD52 group genes. For completeness, the analysis included verified genes that were partially deleted when an unverified gene was precisely deleted during creation of the library. As shown in Table 4, 60 of the non-RAD52 group genes could be classified into groups with shared functions. A large number (14) have previously been linked to transcription regulation. Many others are known to be involved in DNA metabolism, affecting sister chromatid cohesion, histone and chromatin structure, nuclease processing of DNA and chromosome segregation. Thus, most of the genes have functions that could potentially affect DNA repair. Several genes have known roles in DNA replication or repair, including *EXO1*, *SAE2*, *RAD5*, *MMS2*, and *MMS4*[17,44-47]. Several other genes encode mitochondrial proteins, which may be an indication that repair of this genome is compromised in some mutants, though other explanations are possible.

**Table 4:** Cellular functions/processes affected by genes required for resistance to EcoRI

**Function or process**	**Genes**
Sister chromatid cohesion	*CTF4, CTF8, DCC1, HTL1*
Histone modification/remodeling	*ARP5*, *EAF1, GCN5, RTT109(REM50)*^*b*^*, SPT10, UBP8*
Nuclease processing of DNA	*EXO1, MMS4, SAE2, YLR235C/TOP3*^*a*^
Chromosome stability/segregation	*BIK1, CGI121, CNM67, DDC1, MMS22*
Transcription regulation	*APQ13*/*NET1*^*a*^*, BUD32, BUR2, CCR4, NOT5, NUP84, RPB9, BUD30/RPC53*^*a*^*, RTF1, SFP1, SPT20, TAF14, UME6, YML009W-B/SPT5*^*a*^
RNA processing/modification	*CDC40*, *LRP1, LSM7, TRM9, TSR2/YLR434C*^*a*^*, YDR433W/NPL3*^*a*^
Protein posttranslational modification	*AKR1, BCK1, CAX4, MMS2, OCH1, RAD5, UBR1,*
	*YML012C-A/UBX2*^*a*^
Cell membrane/cell wall	*CIS3, HSP150, RVS161, SAM37, VMA7, VPH2/YKL118W*^*a*^
Mitochondrial proteins	*ATP2, IMG2, MCT1, YGL218W/MDM34*^*a*^*, MRPS35, RSM7/YJR114W*^*a*^*, SCO1*

^a^ Gene names separated by a forward slash indicate deletions within two overlapping open reading frames. Functions and processes are described for the verified gene only. Source: The Saccharomyces Genome Database.

^b^ The *RTT109* gene is frequently referred to as *REM50* in the literature.

High-throughput studies using yeast 2-hybrid screens, affinity capture coupled with mass spectrometry and other physical methods have pointed to extensive protein:protein interactions among the eight RAD52 group proteins that are required for resistance to EcoRI (Table 5). Each protein physically interacts with one or more other proteins in the RAD52 group and also with other proteins involved in replication and repair. Interestingly, the proteomics studies have also provided evidence that many of the proteins encoded by the non-RAD52 group genes interact with each other and with known DNA repair proteins. Forty-one of the non-RAD52 group proteins display such interactions, in some cases involving many different proteins (Table 5). For example, Gcn5 is proposed to interact with 6 of the other non-RAD52 group proteins and also with Rad59, providing a link to DSB repair pathways. Names of interacting proteins that affect EcoRI resistance are underlined in the table.

**Table 5:** Physical interactions among proteins required for efficient repair of EcoRI-induced DSBs*

**Name**	**Interacting proteins**	**Name**	**Interacting proteins**
*RAD52 group:*			
Rad50	Mre11, Xrs2, Dun1	Rad55	Rad51, Rad57, Mec1, Rad53
Rad51	Rad52, Rad54, Rad55, Rad57,		Rtt107, Srs2
	Rad59, Rdh54, Mlh1, Rad23,	Rad57	Rad51, Rad55, Rad24, Rtt107,
	Rfa1, Saw1, Sgs1, Srs2		Srs2
Rad52	Bur2, Rad51, Msh6, Rfa1, Rfa2,	Mre11	Rad50, Xrs2, Dmc1, Dna2,
	Rfa3, Saw1, Slx5		Dnl4, Msh5, Sgs1, Srs2, Yku80
Rad54	Rad51, Mus81	Xrs2	Rad50, Mre11, Lif1
*Non-RAD52 group:*			
Adk1	Bck1, Sir2	Net1	Cac2, Rad53, Sir2
Akr1	Gcn5, Dun1	Not5	Ccr4
Arp5	Taf14	Npl3	Bur2, Spt5
Bck1	Adk1, Lip5	Rad5	Pol30, Rad18, Rev1, Srs2
Bud32	Cgi121	Rem50	Asf1, Pol30
Bur2	Npl3, Rad52, Rfa1, Rfa2	Rpb9	Spt5, Taf14
Ccr4	Not5, Ubr1	Rpl12b	Rpl31a, Rpl39
Cgi121	Bud32, Srs2	Rpl31a	Rpl12b, Rpl39
Cnm67	Mlp2	Rpl39	Rp12b, Rpl31a
Ctf4	Mms22	Rsm7	Mrps35
Ctf8	Dcc1	Rtf1	Spt5
Dcc1	Ctf8	Rvs161	Gcn5
Ddc1	Mec3, Rad17, Rad52, Rad53, Rev7	Sae2	Sir3, Srs2
Gcn5	Akr1, Mms4, Rvs161, Srv2, Spt20	Sfp1	Asf1
	Ubp8, Rad59	Spt5	Npl3, Rpb9, Rtf1, Taf14
Gnd1	Ubr1	Spt20	Gcn5, Ubp8
Lip5	Bck1	Srv2	Gcn5
Mms2	Pol30	Taf14	Arp5, Rpb9, Spt5, Mus81
Mms4	Gcn5, Mus81, Rad27	Top3	Dna2, Sgs1
Mms22	Ctf4, Mms1	Ubp8	Gcn5, Spt20, Sir3
Mrps35	Rsm7	Ubr1	Ccr4, Gnd1, Rad6, Srs2

* Includes proteins predicted to interact physically plus proteins that are shared components of a multisubunit complex. Underlined names indicate proteins encoded by genes that are required for resistance to EcoRI overexpression. Other interacting proteins have previously been linked to DSB repair and/or damage-inducible checkpoints. Associations were taken from the Saccharomyces Genome Database.

Many yeast proteins are conserved in lower and higher eukaryotes. To examine conservation of the genes identified in this work, homologies of the protein products of the 81 EcoRI^s^ loci were compared to proteins from three actively studied genomes of higher eukaryotes: human, mouse, and rat (Additional file 2: Table S2). The analysis was conducted using the BLASTp sequence alignment program at the National Center for Biotechnology Information (NCBI), which produces homology scores that are sorted based on “e-values”. Scores with higher negative exponents, e.g., e^-150^, indicate greater sequence homology, while smaller exponents indicate weak or no homology. Analysis of the sequences of the eight yeast RAD52 group proteins revealed that seven of the polypeptides have strong homology with mammalian proteins, displaying e-values as high as e^-169^. With the exception of Xrs2, scores ranged from e^-5^ to e^-169^. Xrs2 is known to be an ortholog of the human protein Nbs1, but its sequence shows only weak similarity to Nbs1 in short regions at the amino and carboxy termini [48].

Forty-five of the other proteins exhibited moderate or strong homology (e^-4^ or better) to proteins from all three mammalian genomes. Strongest homologies were seen with Gnd1, Top3 and Atp2, which displayed scores of e^-158^, e^-149^ and e^-148^, respectively, compared to their human counterparts. Gnd1 regenerates NADPH inside cells, Top3 is a topoisomerase needed during transcription and DNA replication, and Atp2 is a component of a membrane complex important for synthesis of ATP. Each of these reactions corresponds to an essential process common to all organisms and likely explains the unusually high conservation of the proteins. Fifty-two of the 81 proteins (7/8 RAD52 group and 45/73 non-RAD52 group proteins) displayed homology scores of e^-4^ or better, corresponding to 64% of the total. This suggests that most of the yeast genes characterized here have functional counterparts in mammalian cells that are also likely to affect DNA repair processes.

## Discussion

In this study, genes shown in previous genomic screens to be required for resistance to gamma radiation in diploid cells were analyzed to identify those genes that specifically affect repair of site-specific DSBs. This was accomplished by expressing the restriction endonuclease EcoRI in haploid equivalents of each mutant and assessing survival after induction in galactose. Eighty-one mutants were identified as sensitive to EcoRI expression, including eight known RAD52 group mutants plus seventy-three others. A few genes were only tested in *MATa* cells because the *MATα* library versions grew too poorly to be evaluated. This observation, in conjunction with others such as our finding that one of the *MATα* strains (*cdc40*) was Ura^+^, indicate that a small fraction of the haploid library mutants contain uncharacterized mutations in other genes. Such secondary mutations can confound results of genome-wide screens, a concern that has been noted before [19]. Sensitivity to EcoRI expression was confirmed in both *MATα* and *MATa* strains for most mutants in the current study, however, indicating that the results observed using one haploid library were largely reproducible in the other library.

Among RAD52 group mutants, *rad50*, *rad51*, *rad52*, *rad54*, *rad55*, *rad57*, *mre11* and *xrs2* strains were EcoRI^s^, but *rad59*, *rdh54* and *rad61* cells were not. These results, in conjunction with our observation that NHEJ mutants such as *yku70*, *dnl4* and *nej1* cells were not sensitive, indicate that homologous recombination is the most critical pathway in the S288c strain background used to construct the libraries. The resistance of *rad59*, *rdh54* and *rad61* cells is consistent with the more specialized roles of Rad59 and Rdh54 in recombination and the possible major function of Rad61 in sister chromatid cohesion [15,20,49].

The eight RAD52 group mutants were sensitive to every clastogen tested, including bleomycin, MMS and gamma radiation. Among the other 73 mutants, 51 exhibited reduced survival after exposure to bleomycin and 47 mutants were sensitive to MMS. The pronging survival assays employed here were limited to detection of mutants that showed a consistent reduction of 2 fold or more in colony formation. Thus, modest sensitivities in some mutants may not have been detectable.

Although each of the eight RAD52 group mutants was sensitive to gamma radiation as expected, a surprisingly small fraction of the other EcoRI^s^ mutants exhibited this phenotype (32/73). Thus, each of the non-RAD52 group mutants was found to be gamma-sensitive as diploids in previous genomic screens [19,27,28,32], but most of them were not detectably sensitive as haploids in the current study. In the previous study by Bennett *et al.*, diploid mutants were categorized as gamma sensitive if there was (a) reduced survival in pronging assays or (b) a slow recovery from gamma-induced damage (producing small colonies) even if the number of surviving colonies was similar to WT cells [27,28]. In the screen by Game *et al.*, [19], a very different method was utilized involving irradiating pools of diploid deletion library strains, purifying and amplifying DNA from the surviving cells, and using hybridization methods to identify mutants that had reduced abundance in the culture due to cell killing. This latter study detected 33 strongly radiation-sensitive mutants, 22 of which were also identified in the report by Bennett *et al.* In the current study using haploid cells, mutants were only scored as sensitive in semi-quantitative pronging assays if survival was reduced, i.e., the number of surviving colonies on plates was reproducibly decreased by ≥ 2-fold. These differences in methodology and classification are likely to explain many of the differences in radiation sensitivities observed between haploid and diploid strains. However, in at least some cases the differences may be a characteristic of the strains: *srs2* and *rdh54* mutants are known to be radiation sensitive as diploids, but not as haploid cells, and other mutants may share this phenotype [49,50].

Seventeen of the EcoRI^s^ mutants contained deletions of an ORF that overlapped one or more other ORFs. At only one locus, containing the divergent and overlapping genes *MMS4* and *YBR099C*, did the library collection contain separate mutants containing precise deletions of each overlapping ORF. Among the 15 other loci, 5 verified genes that were completely deleted in the library strains (*EAF1*, *GND1*, *RSM7*, *TSR2* and *VPH2*) overlapped unverified ORFs and 10 unverified ORFs whose coding regions were deleted overlapped verified genes (*NET1*, *RPL39*, *RPC53*, *YKL075C*, *RPL12B*, *NPL3*, *MDM34*, *TOP3*, *SPT5*, and *UBX2*). Many of the unverified ORFs are likely to represent nonfunctional genes, so at most loci only one of the overlapping genes is transcribed and translated *in vivo*. However, it is possible that both genes are functional at some of the overlapping regions: a search of the sequences stored at the Saccharomyces Genome Database revealed that 13 yeast chromosomal loci contain overlapping protein-coding genes that are each verified (*ADF1/FYV5, ATG29/SET6, AUA1/WWM1, BUD5/MATα2, CTF19/IRC15, CWC25/VPS75, DCR2/VPS38, EMI1/GRX2, HUR1/PMR1, IMO32/NAG1, NKP2/TAD3, PRP38/SMD1* and *VAM10/VPS5*) and a region on chromosome V contains 3 verified genes whose coding regions overlap each other (*BUD25, FAA2* and *HEM14*).

Among the ten mutants containing a deleted unverified ORF that overlapped a verified gene, it was possible that the sensitivity phenotypes were not caused by the loss of the putative gene that was precisely deleted to create the library strain. Instead, truncation of the overlapping gene could have generated the sensitivity. Tests using seven mutants containing precise deletions of the coding regions of overlapping verified genes revealed that two of them, *rpl39* and *npl3*, were strongly EcoRI^s^, and five others were not. It is therefore possible that the phenotypes of *bud19* and *ydr433w* deletion mutants are actually caused by inactivation of the overlapping *RPL39* and *NPL3* genes, respectively. Additional work will be required to confirm this conjecture and to determine the source of the DNA damage sensitivities in the other mutants containing deletions affecting two or more overlapping ORFs.

Forty-one of the non-RAD52 group genes have previously been linked to nuclear processes such as transcription, nuclease processing of DNA, histone modification, chromosome segregation and sister chromatid cohesion. Some of the genes are already known to affect repair of DSBs. These include *EXO1* and *SAE2*, involved in nuclease resection of DSBs during homologous recombination, and *MMS4*, which encodes a subunit of the Mms4-Mus81 endonuclease involved in cleavage of branched DNA structures [15,23,46,47]. Detection of mutants affecting mitochondria-associated proteins suggests that repair of this organelle’s genome may be compromised in some mutants. Six of the 7 mitochondrial mutants were also sensitive to MMS and/or bleomycin, supporting the idea that a defect in repair of damage to DNA is the main cause of sensitivity in the mutants. Recent studies have demonstrated that many DNA and RNA processing enzymes utilize iron-sulfur clusters, which are synthesized primarily in the mitochondria, indicating a possible source of the sensitivity [51]. The potential roles of several of the other genes in DNA repair, such as those known to affect cell membrane architecture, remains unclear.

RAD52 group proteins exhibit physical interactions with each other and with many other proteins involved in DNA replication and repair (Table 5). Forty-one of the non-RAD52 group proteins have previously been shown to interact with at least one other protein affecting DNA damage resistance. Some proteins, such as Ddc1 and Gcn5, exhibit multiple associations with both RAD52 group proteins and with other repair proteins. These linkages to known repair proteins imply that some of the proteins may have direct roles in DNA repair. Our observation that 64% of the proteins exhibit moderate or strong homology to mammalian proteins suggests further that many of the functions identified in yeast cells will be conserved in higher eukaryotes.

Investigation of the clastogen sensitivities of the mutants characterized in this study revealed unexpected variability. For example, among the 73 non-RAD52 group mutants that were EcoRI^s^, only 19 strains were sensitive to all three other clastogens: MMS, bleomycin and gamma radiation. The remaining strains exhibited mixed sensitivity phenotypes (Figure 3). Most of the mutants were sensitive to MMS and bleomycin, but only 32 were killed by gamma radiation. Since the radiation survival studies involved a single brief exposure and the other three damaging treatments were applied continuously during colony formation on plates, it was possible that the gamma-resistant mutants were only sensitive to agents that generate damage continuously throughout the cell cycle. To test this idea, survival of gamma-resistant and gamma-sensitive mutants was tested after a single brief exposure to the direct DNA-damaging agent bleomycin. All of the radiation-resistant and radiation-sensitive mutants displayed similar, modest sensitivities to a brief exposure to bleomycin, suggesting that continous exposure was not the key to their sensitivities. Although the cells were exposed to bleomycin for a short time (30 min) and were washed extensively afterward, it is possible that some of the drug persisted inside the cells for more than one cell cycle. Such persistence would increase the difficulty of making comparisons between effects caused by radiation versus chemical agents.

All of the mutants characterized in this study have been shown to be radiation-sensitive as diploids (with caveats discussed above) and each mutant was also sensitive to *in vivo* expression of EcoRI, an endonuclease that specifically induces site-specific, cohesive-ended DSBs in DNA. However, over half of the non-RAD52 group mutants were not radiation sensitive as haploid cells. Furthermore, the haploid strains exhibited variable sensitivities to the chemical clastogens MMS and bleomycin (Figure 3). This phenotypic variation, in conjunction with variability in sensitivities seen in other studies assessing the effects of multiple chemicals on yeast cell growth e.g., ref. [31], indicate that caution is needed in interpretation of such experiments.

## Conclusions

In this study a large number of genes were identified that are essential for survival of haploid yeast cells when EcoRI endonuclease is expressed *in vivo*. Mutant phenotypes observed in strains of one haploid library were largely confirmed upon testing of the equivalent mutants from another library. Many library mutants contained deletions of a single putative gene that truncated another overlapping gene; experiments indicated that phenotypes observed in such mutants could be caused, in some cases, by alteration of the overlapping gene. Many of the genes affecting EcoRI sensitivity have previously been linked to DNA and RNA metabolism and several are active in processes known to affect DSB repair, including nucleosome remodeling, sister chromatid cohesion, and DNA damage responsive cell cycle checkpoints. Determining which of the genes are directly involved in DSB repair pathways will require application of specific *in vivo* assays for recombination and NHEJ and direct physical measurements of DSB repair.

## Methods

### Yeast strains and plasmids

Haploid *MATa* and *MATα* yeast deletion strain libraries were obtained from Open Biosystems. The libraries were constructed primarily in yeast strains BY4741 (*MATa his3Δ1 leu2Δ0 met15Δ0 ura3Δ0*) and BY4742 (*MATα his3Δ1 leu2Δ0 lys2Δ0 ura3Δ0*) [52]. One of the RAD52 group mutants in the *MATα* library, expected to contain an *mre11Δ::kanMX* (G418^r^) allele, did not exhibit sensitivity to MMS or bleomycin as expected. A new *MATα mre11* strain was constructed in the BY4742 background by PCR-mediated gene disruption deletion using pFA6MX4 [53] to create YLKL834 (*MATα his3Δ1 leu2Δ0 lys2Δ0 ura3Δ0 trp1::hisG mre11Δ::G418*). Plasmids used in the study included pRS316 (*CEN/ARS URA3*) [54] and YCpGal:RIb (*CEN/ARS URA3 GAL1p::EcoRI*) [33,34].

### Yeast growth media

D-(+)-galactose, D-(+)-glucose, raffinose, plate agar, and amino acids were purchased from Sigma-Aldrich Chemical Co. Difco bacto peptone, bacto yeast extract, bacto tryptone, yeast nitrogen base, and LB broth mix were purchased from Becton Dickinson Microbiological Systems. For general, non-selective growth, yeast cells were grown on YPDA media (1% bacto yeast extract, 2% bacto peptone, 2% glucose, 2% bacto agar, 0.001% adenine). For plasmid selection, yeast cells were grown on synthetic media with drop-out mix (0.17% yeast nitrogen base without amino acids, 2% glucose, 2% bacto agar, and all essential amino acids minus the amino acids used for selection). For the EcoRI assays, yeast cells were grown on synthetic media minus uracil containing 2% raffinose or 3% galactose. Plates with methyl methanesulfonate (MMS) were prepared using synthetic media or YPDA supplemented with aliquots of a stock solution of 11.2 M MMS (Sigma-Aldrich). Bleomycin plates were made using synthetic media plus aliquots from a stock solution of 0.5 mg/ml bleomycin (EMD Chemicals, Inc.) in ddH_2_O. Transformation of plasmid DNAs into yeast cells was performed using a rapid lithium acetate/DMSO-based transformation protocol [55].

### Double-imprint replica-plating and pronging survival assays

To develop an assay that demonstrated EcoRI killing of known DSB repair mutants, patches of mutant and WT cells containing plasmids were grown on 2% raffinose minus uracil (Raff-Ura) synthetic plate media for 2–3 days and replica-plated to a new Raff-Ura plate. This plate was immediately used as a new master plate to replica-plate cells to Raff-Ura and galactose minus uracil (Gal-Ura) plates. These “double-imprint” replica plates were grown at 30°C for 2–4 days. The EcoRI double imprinting assay was then applied to the collection of haploid *MATα* library mutants used initially in the study.

Mutants exhibiting EcoRI sensitivity in the initial screen were subsequently tested more quantitatively using dilution pronging survival assays. Yeast cells were harvested from Raff-Ura plates into water, diluted 1/40, sonicated for 10 seconds, and quantitated using a hemocytometer in conjunction with a phase contrast microscope. A total of 2 × 10^7^ cells were then added to a 96-well mictrotiter dish, followed by serial 5-fold dilutions and pronging onto selective plates. Pronged cells were grown at 30°C for 4–7 days, long enough to ensure detection of slow-growing colonies. Mutants were classified as resistant to EcoRI (R), moderately sensitive (S), or strongly sensitive (SS) based on counting the number of colonies present at the highest dilutions. Moderately sensitive mutants exhibited < 25-fold killing relative to wildtype cells on plates with galactose, i.e., colony number was reduced by less than 2 columns relative to wildtype cells expressing the EcoRI plasmid. Strongly sensitive mutants exhibited ≥ 25-fold killing on plates with galactose, which is equal to or more than two full columns of growth less than in wildtype cells. The sensitivity of the assay was limited to detection of mutants that consistently exhibited 2–3 fold fewer colonies than wildtype cells.

For gamma radiation survival studies, overnight yeast cultures were diluted 1:10 in fresh YPDA broth and grown at 30°C for 4 h. Cells were serially diluted 5-fold in ddH_2_O and irradiated at 0, 30 and 60 krads in 96-well microtiter plates using a ^137^Cs source. The cells were pronged in quadruplicate to YPDA plates immediately after irradiation and incubated at 30°C for 3–5 days.

### Single-hit analysis of bleomycin sensitivities

For experiments involving a single exposure to bleomycin, overnight cultures grown in YPDA broth (3 mL) were diluted 1:20 into 600 μL YPDA broth using four cultures for each strain tested. The cultures were shaken vigorously for 2 hrs at 30°C. Cells from each culture were then exposed to 10 μg/mL bleomycin for 30 min at RT. The cells were pelleted and resuspended in YPDA broth without bleomycin three times to wash out the drug. Appropriate dilutions were spread onto YPDA plates and incubated at 30°C for 3–4 days.

### Bioinformatics analysis

For analysis of homologies among proteins, sequences were compared utilizing the Saccharomyces Genome Database (SGD) (http://www.yeastgenome.org) and the Basic Local Alignment Search Tool (BLASTp) program at the National Center for Biotechnology Information. Yeast protein sequences initially retrieved from the SGD were used in searches against each of the three genomes, human (*Homo sapiens*), mouse (*Mus musculus*), and rat (*Rattus norvegicus*) using the appropriate RefSeq protein database. Protein:protein associations were similarly assessed using sources located within and linked from the SGD database. Physical interactions based on 2-hybrid screens, affinity capture with mass spectrometry and related methods were evaluated, but associations inferred based only on co-localization experiments were not used. Genetic interactions were also not included in the analysis. For assessment of cellular processes associated with each protein, the Locus Summary gene descriptions and the “’Biological Process” associations described in the Gene Ontology (GO) resources at the SGD were analyzed. Identification of verified genes whose reading frames overlap was accomplished using the YeastMine tool at the SGD in conjunction with the “All overlapping genes” template and the program constraint set to display verified genes only.

## Abbreviations

DSB: Double-strand break; NHEJ: Nonhomologous end-joining; HR: Homologous recombination; ssDNA: Single-stranded DNA; MMS: Methyl methanesulfonate; EcoRIs: EcoRI-sensitive; Raff-Ura: Raffinose media minus uracil; Gal-Ura: Galactose media minus uracil; ORF: Open reading frame; GO: Gene ontology; SGD: Saccharomyces genome database.

## Competing interests

The authors declare that they have no competing interests.

## Authors’ contributions

JSM, SS and JDT performed most library screening and mutagen sensitivity experiments involving EcoRI, MMS and bleomycin as well as the bioinformatics analyses of genes and proteins. Tests of single-hit bleomycin sensitivities and analyses of overlapping reading frames were performed by BAS and TNN. JWW and MAR screened library strains for gamma radiation resistance. All authors read and approved the final manuscript.

## Supplementary Material

Additional file 1: Table S1 *MAT*α library mutants resistant to *in vivo* expression of EcoRI. Click here for file

Additional file 2: Table S2 Many of the yeast proteins linked to DSB repair have moderate or strong homology to human and animal proteins. Click here for file
