# Supplementary material for: A multistep genomic screen identifies new genes required for repair of DNA double-strand breaks in Saccharomyces cerevisiae
Source: BMC Genomics. 2013 Apr 15;14:251. doi: 10.1186/1471-2164-14-251 (PMC3637596; doi:10.1186/1471-2164-14-251)
Supplement: Additional file 1: Table S1 — MATα library mutants resistant to in vivo expression of EcoRI. [file 1471-2164-14-251-S1.docx]

Table S1. *MATα* library mutants resistant to *in vivo* expression of EcoRI

*ada2 apn1 ard1 arp8 asf1*

*asm4 atp4 bdf1 bem1 bfr1*

*bmh1 bre1 chl1 ckb1 clc1*

*deg1 dhh1 dia4 doc1 dot1*

*eap1 efg1 eos1 est1 fab1*

*fil1 fun12 glo3 gon7 gos1*

*gpx2 grr2 her2 hmo1 hof1*

*hpr1 idp1 irc4 iwr1 jem1*

*ldb7 lge1 lhs1 loc1 map1*

*mbp1 mdj1 mdm10 mdm20 mec3*

*mnn11 mrp10 mrpl31 mrt4 mtc7*

*mus81 myo4 nab6 nat1 nat3*

*not4 npl6 nup120 nup133 nup170*

*nup188 pat1 pfd1 pfk2 pho2*

*plc1 pol32 pre9 pso2 rad1*

*rad6 rad9 rad10 rad17 rad18*

*rad24 rad27 rad59 rad61 rai1*

*rdh54 ref2 rim1 rkm1 rmd6*

*rpl20a rpl34b rsa1 rsc1 rsc2*

*rvs167 sac6 scp160 sgs1 slx8*

*smi1 srb5 srs2 sse1 ssz1*

*tda5 tho2 thr1 tif4631 tps1*

*tps2 tup1 ubc13 vid31 vps33*

*vps65 yaf9 ybr100w ycl007c ydj1*

*ydl041w ydr532c yjl193w ylr358c yml009c-a*

*ypl066w ypl071c zuo1*
