# Supplementary material for: A multistep genomic screen identifies new genes required for repair of DNA double-strand breaks in Saccharomyces cerevisiae
Source: BMC Genomics. 2013 Apr 15;14:251. doi: 10.1186/1471-2164-14-251 (PMC3637596; doi:10.1186/1471-2164-14-251)
Supplement: Additional file 2: Table S2 — Many of the yeast proteins linked to DSB repair have moderate or strong homology to human and animal proteins. [file 1471-2164-14-251-S2.docx]

Table S2. Many of the yeast proteins linked to DSB repair have moderate or strong homology to

human and animal proteins (e-value ≤ 10^-4^)^a^

**RAD52 Accession Accession**

**Group: Protein Human No. Mus/Rattus^b^ Protein Human No. Mus/Rattus^b^**

Rad50  *3e^-67^ NP_005723.2 1e^-136^/1e^-66^* Rad55 *5e^-5^ NP_002868.1 1e^-6^/3e^-6^*

Rad51 *3e^-169^ NP_002866.2 8e^-169^/2e^-168^* Rad57 *2e^-20^ NP_005423.1 6e^-19^/3e^-17^*

Rad52 *2e^-48^ NP_602296.2 1e^-49^/4e^-49^* Mre11 *7e^-129^ NP_005582.1 6e^-131^/9e^-129^*

Rad54 *3e^-153^ NP_003570.2 2e^-148^/4e^-154^* Xrs2 *(2.6) - (4.2/2.6)*

**This**

**Study:**  Gnd1^c^  *5e^-158^ NP_002622.2 2e^-178^/4e^-178^* Arp5 *8e^-39^ NP_079131.3 2e^-38^/7e^-38^*

Top3  *4e^-149^ NP_004609.1 5e^-147^/1e^-96^* Bud32 *5e^-34^ NP_291028.3 1e^-36^/4e^-37^*

Atp2 *4e^-148^ NP_001677.2 3e^-155^/2e^-167^* Vma7 *5e^-34^ NP_004222.2 2e^-33^/2e^-33^*

Lip5 *5e^-136^ NP_006850.2 2e^-138^/9e^-140^* Rpb9 *1e^-30^ NP_006224.1 9e^-31^/8e^-31^*

Gcn5  *2e^-94^ NP_003875.3 4e^-94^/1e^-94^* Ubr1 *2e^-28^ NP_056070.1 6e^-32^/2e^-31^*

Ccr4 *7e^-94^ NP_056270.2 3e^-90^/3e^-90^* Lsm7 *1e^-23^ NP_057283.1 2e^-23^/8e^-24^*

Adk1 *4e^-90^ NP_037543.1 2e^-91^/3e^-91^* Cax4 *5e^-23^ NP_065171.2 1e^-22^/3e^-16^*

Cdc40 *2e^-87^ NP_056975.1 5e^-88^/4e^-86^* Rtf1 *3e^-20^ NP_055953.3 3e^-20^/8e^-20^*

Rpl12b^c^  *3e^-84^ NP_000967.1 1e^-84^/9e^-84^* Cgi121 *1e^-19^ NP_057142.1 2e^-20^/4e^-20^*

Rad5 *1e^-82^ NP_003062.2 2e^-83^/7e^-85^* Ctf4 *5e^-19^ NP_009017.1 1e^-11^/1e^-15^*

Srv2 *1e^-77^ NP_006357.1 2e^-75^/3e^-75^* Rpl39  *1e^-18^ NP_000991.1 8e^-19^/8e^-19^*

Ado1 *3e^-77^ NP_001114.2 2e^-78^/6e^-79^* Rvs161 *3e^-18^ NP_061158.1 5e^-19^/1e^-18^*

Spt5^c^  *6e^-70^ NP_001124297.2 4e^-72^/3e^-72^* Npl3^c^ *4e^-15^ NP_006266.2 3e^-15^/1e^-15^*

Akr1 *3e^-62^ NP_056151.2 1e^-61^/2e^-61^* Nup84 *8e^-14^ NP_065134.1 1e^-12^/1e^-11^*

Ubp8 *5e^-61^ NP_056091.1 2e^-61^/2e^-61^* Sfp1 *3e^-11^ NP_778231.2 1e^-11^/3e^-11^*

Exo1 *1e^-60^ NP_003677.4 1e^-58^/4e^-58^* Taf14 *1e^-9^ NP_004520.2 3e^-9^/3e^-7^*

Bck1 *4e^-60^ NP_002392.2 2e^-60^/6e^-61^* Tsr2^c^ *1e^-9^ NP_477511.1 4e^-10^/2e^-9^*

Trm9 *1e^-50^ NP_630130.2 2e^-52^/5e^-51^* Bik1 *2e^-8^ NP_056069.2 6e^-8^/3e^-8^*

Mms2 *4e^-48^ NP_003341.1 3e^-47^/3e^-47^* Dcc1 *3e^-8^ NP_076999.2 3e^-8^/4e^-6^*

Sco1 *6e^-45^ NP_004580.1 8e^-46^/4e^-46^* Rpc53^c^ *5e^-8^ NP_001713.2 5e^-9^/3e^-8^*

Rpl31a *3e^-43^ NP_000984.1 2e^-43^/2e^-43^* Img2 *2e^-6^ NP_004918.1 2e^-7^/2e^-7^*

Not5 *5e^-43^ NP_055331.1 3e^-43^/3e^-43^* Eaf1 *1e^-4^ NP_056224.3 2e^-4^/2e^-4^*

Sam37 *5e^-4^ NP_002446.2 9e^-5^/4.3*

^a^ Values shown are exponents, e.g., 3e^-65^ is an e-value of 3x10^-65^. Larger negative exponents indicate stronger homology.

NCBI RefSeq Accession Numbers are shown for the top-scoring human proteins. ^b^ Mus, mouse; Rattus, rat. ^c^ These proteins

are encoded by genes whose coding regions overlap one or more other open reading frames. The protein product of the

verified gene was used for each BLAST search.
